# Subcutaneous Implantable Cardioverter-defibrillator Explantation—A Single Tertiary Center Experience

**DOI:** 10.19102/icrm.2022.130407

**Published:** 2022-04-15

**Authors:** Naga Venkata K. Pothineni, Tharian Cherian, Neel Patel, Jeffrey Smietana, David S. Frankel, Rajat Deo, Andrew E. Epstein, Francis E. Marchlinski, Robert D. Schaller

**Affiliations:** ^1^Section of Cardiac Electrophysiology, Division of Cardiovascular Medicine, University of Pennsylvania, Philadelphia, PA, USA

**Keywords:** Explantation, extraction, infection, pacing, subcutaneous implantable cardioverter-defibrillator

## Abstract

The subcutaneous implantable cardioverter-defibrillator (S-ICD) is an appealing alternative to transvenous ICD systems. However, data on indications for S-ICD explantations are sparse. The objective of this study was to assess the incidence and indications for S-ICD explantation at a large tertiary referral center. We conducted a retrospective study of all S-ICD explantations performed from 2014–2020. Data on demographics, comorbidities, implantation characteristics, and indications for explantation were collected. A total of 64 patients underwent S-ICD explantation during the study period. During that time, there were 410 S-ICD implantations at our institution, of which 53 (12.9%) were explanted with a mean duration from implant to explant of 19.7 ± 20.1 months. The mean age of the patients at explantation was 44.8 ± 15.3 years, and 42% (n = 27) were women. The indication for S-ICD implantation was primary prevention in 58% and secondary prevention in 42% of patients, respectively. The most common reason for explantation was infection (32.8%), followed by abnormal sensing (25%) and the need for pacing (18.8%). Those who underwent S-ICD explantation for pacing indications were significantly older (55.7 ± 13.6 vs. 42.3 ± 14.6 years, *P* = 0.005) with a wider QRS duration (111 ± 19 vs. 98 ± 19 ms, *P* = 0.03) at device implantation compared to patients who underwent explantation for other indications. The incidence of S-ICD explantation in a large tertiary practice was 12.9%. While infection was the indication for one-third of the explantations, a significant number of explantations were due to sensing abnormalities and the need for pacing. These data may have implications for patient selection for S-ICD implantation.

## Introduction

The implantable cardioverter-defibrillator (ICD) represents a major advance in the prevention of sudden cardiac death. Since their development, the implantation of transvenous ICDs (TV-ICDs) has become the standard of care for the primary and secondary prevention of sudden cardiac death.^1^ Long-term use of TV-ICD, however, has led to a rise in device-related complications, such as lead fracture and infections,^2,3^ and the introduction of the totally subcutaneous ICD (S-ICD) heralded the appearance of an attractive alternative. Since the pivotal clinical trials establishing the safety and efficacy of S-ICD,^4,5^ this technology is no longer reserved for only patients at high risk for TV-ICD complications. Increased utilization has also led to a concomitant rise in S-ICD–associated complications, occasionally necessitating explantation. However, real-world data on S-ICD removal are sparse. We sought to assess the incidence and indications for S-ICD explantations at a large tertiary referral center.

## Methods

### Study design

We conducted a retrospective study of all patients who underwent S-ICD explantation at the Hospital of the University of Pennsylvania from January 2014 to December 2020. All patients provided written informed consent for the procedure and for their anonymized medical information to be included in research studies, and this study was approved by the institutional review board. Data on demographics, comorbidities, implant characteristics, and indications for explantation were collected from electronic health records. Indication for implantation, electrocardiogram characteristics, and shock impedance during defibrillation threshold (DFT) testing at initial implant were obtained. Patients who underwent S-ICD implantation at our institution and later underwent device removal at a different institution were identified by querying the manufacturer’s database for device status. Details of indications for device removal at other institutions were obtained through the manufacturer’s records, a review of electronic health records, and by telephone calls to patients when needed. Data on clinical management post–S-ICD explantation were also obtained.

### Explantation procedure

S-ICD explantation was performed in the electrophysiology laboratory under general anesthesia with patients prepped and draped in a similar fashion as that during implantation. Incisions were made at the inframammary and xiphoid regions with the addition of a superior parasternal incision for patients who originally underwent the 3-incision insertion technique. In cases of infection, pockets were fully debrided and closed. The placement of a surgical drain was at the discretion of the operator.

### Statistical analysis

Categorical variables are summarized with frequencies (percentages) and continuous data are summarized using mean and standard deviation values. Categorical variables were compared using a chi-squared test or Fisher’s exact test. Continuous variables were compared using Student’s *t*-test. *P* < 0.05 was considered to be statistically significant. All analyses were performed with SPSS version 11.0 (IBM Corporation, Armonk, NY, USA).

## Results

A total of 410 patients underwent S-ICD implantation at our institution during the study period. First-generation model 1010 pulse generators (Boston Scientific, Marlborough, MA, USA) were implanted in 88 (21.5%) patients, while second- (model A209) and third-generation (model A219) pulse generators (Boston Scientific) were implanted in the remaining 322 (78.5%) patients. Among patients who underwent S-ICD implantation at our institution, a total of 53 patients (12.9%) underwent subsequent explantation. An additional 11 S-ICD explants were performed on patients who underwent implantation at other institutions, for a total of 64 S-ICD explantations at our center during the study period. All S-ICD leads were removed with simple traction, and there were no complications.

### Patient characteristics

The baseline characteristics of patients who underwent S-ICD explantations are summarized in **Table 1**. The mean age of patients at device explant was 44.8 ± 15.3 years, and 42% were women. The indication for initial implantation was primary prevention in 58% (n = 37) and secondary prevention in 42% (n = 27) of patients. The mean QRS duration and left ventricular ejection fraction at implantation were 100.1 ± 19.5 ms and 40.9% ± 19.5%, respectively. The mean shock impedance at implantation was 68.4 ± 24.4 Ω. The mean duration from S-ICD implant to explant was 19.7 ± 20.1 months.

### Indications for subcutaneous implantable cardioverter-defibrillator explantation

**Figure 1:**
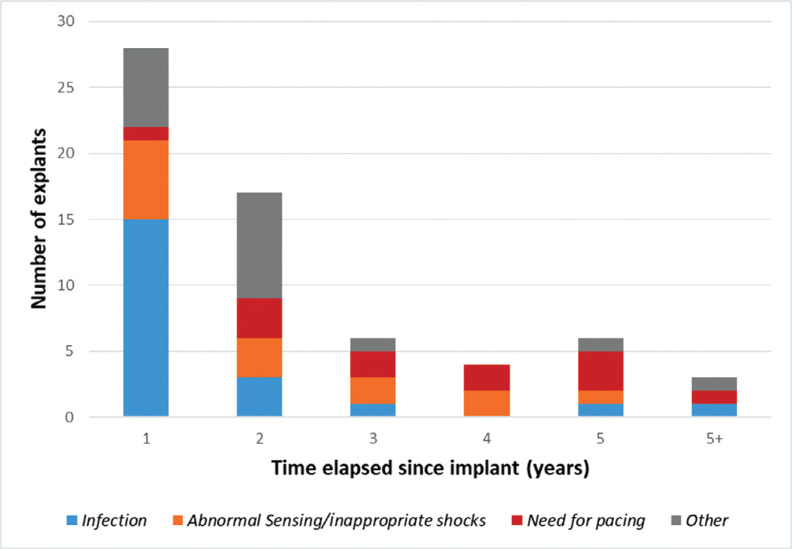
Subcutaneous implantable cardioverter-defibrillator explantations over time arranged by explant indication.

The indications for S-ICD explant are summarized in **Table 2**. The most common reasons for explantation were pocket infection (33%), followed by inappropriate shocks (19%) and the need for pacing (19%). Other indications for S-ICD explantation included progression to heart transplantation or left ventricular assist device (LVAD) implantation, inappropriate device function at pulse generator change, and patient discomfort. The proportion of S-ICD explants by indication over time is shown in **Figure 1**. A significant number of explants (n = 28, 44%), including the majority performed for infection (71%), occurred within the first year of implantation, with a decline in frequency thereafter. Explantation for abnormal sensing remained relatively constant with time, while the proportion of S-ICD explants for a pacing indication increased with longer implant duration.

### Subcutaneous implantable cardioverter-defibrillator system infection

A total of 21 patients underwent S-ICD explantation for device pocket infection. Clinical presentations of these patients included discharge from the incision site, chronic inflammatory reaction over the device site with skin adherence to the generator with or without local erosion, and frank exposure of the device. While the device pocket was the site of infection in the majority of patients, the sub-xiphoid incision was the source of infection in 4 patients. Following device removal, 11 of 21 patients underwent TV-ICD implantation, while 2 patients underwent uncomplicated S-ICD re-implantation after a median of 56 days without recurrent infection. Five patients were lost to follow-up after S-ICD explantation, and 3 patients declined re-implantation.

### Pacing requirement

A total of 12 patients underwent S-ICD explantation and implantation of a transvenous device for a pacing indication. The majority (n = 10) required cardiac resynchronization therapy for progressive heart failure, while 2 patients required pacing for sinus node dysfunction. The baseline and implant characteristics of these patients in comparison to the rest of the cohort are summarized in **Table 3**. Patients who underwent S-ICD explantation for pacing indications were significantly older (55.7 ± 13.6 vs. 42.3 ± 14.6 years, *P* = 0.005) and had a wider QRS duration (111 ± 19 vs. 98 ± 19 ms, *P* = 0.03) at device implant compared to patients who underwent explantation for other indications. There were no statistically significant differences in baseline sinus rates, prevalence of atrial fibrillation, or type of QRS morphology.

### Abnormal sensing/therapy

**Figure 2:**
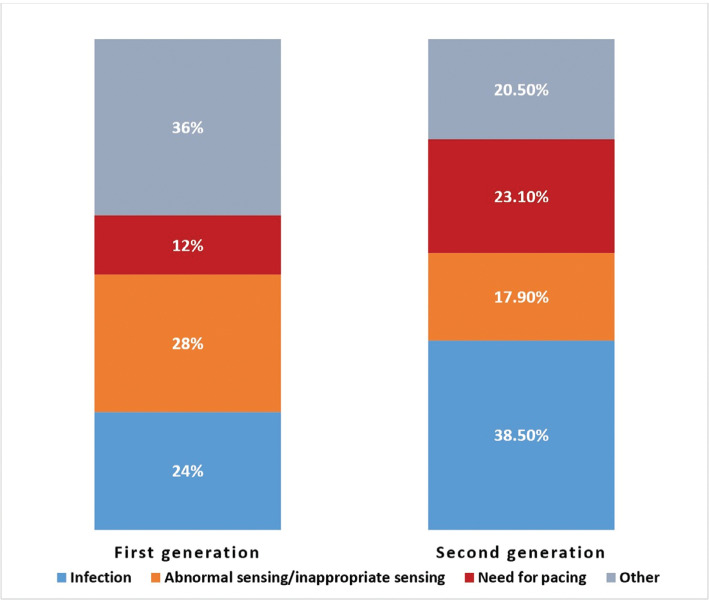
Indications for explantation arranged by generation of subcutaneous implantable cardioverter-defibrillator.

Sixteen patients underwent S-ICD explantation for abnormal sensing or failure of therapy not amenable to reprograming, including changes in the sensing vector and the use of the SMART pass filter (Boston Scientific) and conditional shock zone. Of these, 11 had inappropriate shocks secondary to T-wave oversensing (n = 4), R-wave double counting (n = 3), myopotential oversensing (n = 2), and external noise/artifact (n = 2). All 11 patients underwent implantation of a TV-ICD at the time of explant. One patient underwent explantation at a different institution for inappropriate shocks as per the manufacturer’s records. One patient had oversensing (without inappropriate shocks) due to poor signal-to-noise ratio in all 3 vectors for which the S-ICD was replaced with a TV-ICD. One patient had a prior appropriate shock for ventricular tachycardia, but was later found to have undersensing of ventricular fibrillation by the S-ICD that was detected by an implantable loop recorder for which the S-ICD was replaced with a TV-ICD. Two patients failed DFT testing, leading to replacement of the S-ICD with a TV-ICD; among these, 1 was for repeat DFT testing after a change in the sensing vector, and the other patient failed DFT testing in multiple configurations at the time of the implant after a pocket closure and was subsequently converted to a TV-ICD during the same procedure. The implantation characteristics of patients who underwent S-ICD explantation for abnormalities in sensing and therapy, compared to the rest of the cohort, are summarized in **Table 4**. No significant baseline differences were observed between these 2 groups. The proportion of S-ICD explants for abnormal sensing was similar between the first- and second-/third-generation pulse generators **(Figure 2)**.

## Discussion

In this real-world experience of post-implant S-ICD surveillance from a large tertiary referral center, we report a 12.9% incidence rate of S-ICD system explantation with a mean dwell time of 20 months. While pocket infection was the indication in one-third of cases, abnormal sensing and the need for pacing accounted for >40% of explants, with the need for pacing being the dominant indication after the first year **(Figure 1)**. Patient discomfort, suboptimal device parameters at pulse generator change, and progressive cardiomyopathy requiring LVAD or heart transplantation were other indications. Of note, S-ICD reprogramming and testing of alternative vectors were performed if possible and explantation was used as a last resort.

Previous studies have evaluated similar adverse events and S-ICD explantation rates in prior cohorts. In the pooled analysis of the S-ICD IDE study and EFFORTLESS registry,^5^ a total of 882 S-ICD implants were followed for 3 years, and a total of 108 complications—the majority of which occurred in the first 30 days—were documented. The incidence of S-ICD explantation for a pacing indication was 0.4%. Although not reported, all 25 patients (2.9%) with local infection presumably also underwent explantation. The S-ICD post-approval study evaluated rates of complications in patients who underwent implantation between 2013–2016.^6^ Of the 1,637 procedures, a total of 13 patients (0.08%) underwent S-ICD explantation for infection (8 patients) and failure to defibrillate (5 patients).

The recently reported Prospective Randomized Comparison of Subcutaneous and Transvenous Implantable Cardioverter-defibrillator Therapy (PRAETORIAN) trial randomized 849 patients to TV- and S-ICDs from 2011–2019.^7^ Over a 4-year follow-up period, composite rates of device-related complications and inappropriate shocks were similar in both arms, while the incidence of device-related complications was 5.9% in the S-ICD arm and 9.8% in the TV-ICD arm. The rates of inappropriate shocks in the S-ICD and TV-ICD arms were 9.7% and 7.3%, respectively. Out of a total of 41 inappropriate shocks in the S-ICD arm, 32 (78%) were due to oversensing of cardiac and extra-cardiac signals. Of note, 11 patients in the S-ICD arm who were adjudicated as having appropriate shocks experienced device therapy for oversensing of ventricular arrhythmias below the detection zone. Whether re-adjudication of some of these events as inappropriate shocks would change the primary outcome has been discussed.^8^ A total of 14 (3.3%) patients underwent explantation and crossover to the TV-ICD arm for various indications, including a pacing indication (43%).

Gold et al. recently reported results of the Understanding Outcomes with the S-ICD in Primary Prevention Patients with Low Ejection Fraction (UNTOUCHED) study evaluating rates of inappropriate shocks in a more contemporary primary prevention ICD patient population consisting of 1,111 patients with second- and third-generation S-ICDs from 2015–2018.^9^ Over an 18-month follow-up period, the rate of overall freedom from inappropriate shocks was 95.9%, with a history of atrial fibrillation, non-ischemic cardiomyopathy, or the use of a 2-incision technique being associated with higher rates of inappropriate shocks. Importantly, patients with hypertrophic cardiomyopathy, who have a higher incidence of T-wave oversensing,^10^ were not included. Excluding device inactivation for patient death, a total of 45 patients (4.1%) underwent S-ICD explantation, with the majority of these procedures performed for infection, device malfunction, or progression to LVAD implantation or heart transplantation, reflective of the progression of underlying cardiomyopathy.

Despite increasing awareness of S-ICD device–related complications, real-world data on S-ICD explantation rates are sparse and clinical management of device malfunction such as inappropriate shocks can be challenging. In a series of 108 S-ICD implants, Noel et al. reported a 15.7% (17 patients) incidence of oversensing,^11^ and device explantation was required in 6 patients (5.6%). These rates are similar to our institutional experience and are significantly higher than those reported in larger post-approval studies. In a review of S-ICD events reported to the Manufacturer and User Facility Device Experience (MAUDE) database, Zeitler et al. analyzed a total of 1,604 S-ICD–related complications.^12^ The most common adverse event reported was inappropriate shocks due to oversensing (29%). In this series, a total of 550 inappropriate shocks were reported, of which 69% were due to oversensing. One-third of these instances necessitated system removal and implantation of a TV-ICD. The second most common adverse event was infection, which accounted for 542 reported events, of which 77.5% ultimately underwent system removal. However, this study was limited by the lack of a denominator (number of implants) to assess the true incidence of explantations. In addition, as reporting of events in MAUDE is voluntary, there may have been under-reporting of some complications.

Reasons for differences in overall rates of S-ICD explantation from prior studies are unclear and likely multifactorial, including differences in patient characteristics due to regional institutional referral patterns. Certain centers may have a lower threshold to implant an S-ICD in patient populations who may require the use of anti-arrhythmic medications or catheter-based ablation for arrhythmias that may be amenable to anti-tachycardia pacing. Treatment strategies for pocket infection, impending erosion, or discomfort may also differ in centers, ranging from immediate removal to multiple pocket revisions. Importantly, non-invasive screening for S-ICD candidacy is nuanced, and some providers may accept a borderline result in an effort to avoid transvenous leads. Some patients have borderline indications or are at high risk of requiring pacing over time, and the decision to place an S-ICD in these individuals involves trade-offs that are part of a shared decision-making strategy. Lastly, our real-world data include all iterations of the S-ICD generator and lead as well as implant techniques from original approval to the current day. This included a significant learning curve for electrophysiologists with regard to surgical techniques and anatomical optimization.

Despite the relatively high rate of S-ICD explantation, it remains an appealing alternative to TV-ICDs as it reduces endovascular lead–related complications. In our study, patients who developed the need for pacing tended to be older with wider QRS durations at baseline. Prior studies of TV-ICDs have shown atrioventricular conduction abnormalities and a history of atrial fibrillation to predict the need for pacing,^13^ a finding that was not replicated in our study. However, a QRS cut-off value of 110 ms, above which was associated with a significantly higher degree of S-ICD removal and transition to a TV-ICD, may be considered as a reference guide for future device selection, if confirmed by others.

Two additional factors may affect rates of S-ICD explantations in the future. In a study of 25 patients undergoing S-ICD pulse generator changes, Rudic et al. reported a 20% incidence of defibrillation failure,^14^ possibly related to traditional implantation techniques no longer in vogue. Pocket revision and pulse generator repositioning were corrective in all patients who failed DFT testing. In our study, 1 patient underwent S-ICD explantation for failed DFT testing at generator change. Larger studies evaluating DFT testing at S-ICD generator change may help in clarifying the real-world incidence of these events and the rates of S-ICD explantation for this indication. Second, there were 3 recent U.S. Food and Drug Administration recalls involving S-ICD models A209 and A219 for accelerated battery depletion^15^ and the potential for electrical overstress during delivery of high-voltage therapy,^16^ as well as lead model 3501 for the potential of electrode body fracture distal to the proximal sense ring.^17^ While the recommendation of the manufacturer is to continue routine device follow-up and remote monitoring, these recalls will involve >88,000 patients and may result in an increase in S-ICD explantation.

### Limitations

Our study is limited by its retrospective nature within a single tertiary care center. Data regarding preoperative sensing testing and its impact on explantation rates were not available for analysis. S-ICD technology and device implantation techniques have changed during the study period, which may impact rates of device-related complications that may lead to explantation.

## Conclusions

We report a 12.9% rate of S-ICD explantation in a real-world, contemporary tertiary clinical practice. Infections, abnormal sensing, and the need for pacing were the most common indications for explant. Our study adds to the growing literature assessing the S-ICD complication rates over longitudinal follow-up and determining the incidence of and indications for S-ICD explantations. Further studies are needed to assess rates of S-ICD explantation across various practice settings and identify risk factors that may aid in patient selection. Cost-effectiveness analyses of S-ICDs versus TV-ICDs, considering rates of complications, explantations, and generator changes, might add information to optimal device management strategies.

## Figures and Tables

**Table 1:** Baseline Characteristics of Patients Who Underwent Subcutaneous Implantable Cardioverter-defibrillator Explantation

Baseline Characteristics (n = 64)	
Age at explant, years	44.8 ± 15.3
Women, n (%)	27 (42%)
BMI, kg/m^2^	28.6 ± 6.8
Hypertension, n (%)	33 (52%)
Diabetes mellitus, n (%)	11 (17%)
Coronary artery disease, n (%)	19 (30%)
Renal insufficiency, n (%)	11 (17%)
Atrial fibrillation, n (%)	12 (19%)
Congestive heart failure, n (%)	42 (66%)
Cardiomyopathy, n (%)	50 (78%)
Ischemic, n	10
Non-ischemic, n	
Dilated CM	21
ARVC	5
Congenital	4
Sarcoidosis	4
Peripartum	2
Cardiac masses	2
Non-compaction	1
HCM	1
Indication for implant, n (%)	
Primary prevention	37 (58%)
Secondary prevention	27 (42%)

*Abbreviations:* ARVC, arrhythmogenic right ventricular cardiomyopathy; BMI, body mass index; CM, cardiomyopathy; HCM, hypertrophic cardiomyopathy.

**Table 2:** Subcutaneous Implantable Cardioverter-defibrillator Explanation Indications

S-ICD Explantation (n = 64) Indications	N (%)
**Infection**	21 (32.8%)
**Inappropriate shocks**	12 (18.8%)
**Oversensing (without shocks)**	1 (1.6%)
**Undersensing VF**	1 (1.6%)
**Unsuccessful defibrillation**	2 (3.1%)
**Need for pacing**	
** Cardiac resynchronization**	10 (15.6%)
** Sinus node dysfunction**	2 (3.1%)
**Patient discomfort**	3 (4.7%)
**Heart transplant/LVAD**	7 (10.9%)
**Other**	
** Need for MRI**	1 (1.6%)
** Impedance high at PG change**	1 (1.6%)
** Premature battery depletion**	1 (1.6%)
** Failed DFT testing at PG change**	1 (1.6%)
**No data available**	1 (1.6%)

*Abbreviations:* DFT, defibrillation threshold; LVAD, left ventricular assist device; MRI, magnetic resonance imaging; PG, pulse generator; S-ICD, subcutaneous implantable cardioverter-defibrillator; VF, ventricular fibrillation.

**Table 3:** Implant Data Comparison Between Patients Who Had a Device Explanted for Pacing Indication Versus Other Reasons

	Explant for Pacing Indications (n = 12)	Explant for Non-pacing Indications (n = 52)	*P* value
**Age, years**	55.7 ± 13.6	42.3 ± 14.6	.005
**Atrial fibrillation**	3 (25%)	9 (17%)	.54
**LA size, cm**	4.1 ± 1.0	3.9 ± 0.9	.43
**HR on day of implant, bpm**	70 ± 10	76 ± 16	.26
**QRS duration, ms**	111 ± 19	98 ± 19	.03
**First-degree AV block**	1 (8%)	4 (8%)	.99
**RBBB**	0 (0%)	1 (2%)	.99
**LBBB**	2 (17%)	1 (2%)	.11
**Non-specific intraventricular conduction block**	4 (33%)	14 (26.9%)	.07
**First-generation pulse generators (model 1010)**	3 (25%)	22 (42%)	.94

*Abbreviations:* AV, atrioventricular; HR, heart rate; LA, left atrium; LBBB, left bundle branch block; RBBB, right bundle branch block.

**Table 4:** Implant Data Comparison Between Patients Who Had a Device Explanted for Sensing Issues, Inappropriate Shocks, and Unsuccessful Defibrillation Versus Other Reasons

	Explantation for Sensing Issues, Inappropriate Shocks, Unsuccessful Defibrillation (n = 16)	Explantation for Other Reasons (n = 48)	*P* value
**Age, years**	42 ± 14	50 ± 15	.06
**LVEF**	42% ± 18%	40% ± 20%	.76
**LVIDd, cm**	5.2 ± 0.9	5.5 ± 1.1	.38
**Atrial fibrillation**	2 (13%)	10 (21%)	.96
**QRS duration, ms**	96 ± 14	101 ± 21	.37
**Shock impedance, Ω**	69 ± 30	68 ± 22	.85
**First-generation pulse generators**	8 (50%)	17 (35%)	.30

*Abbreviations:* LVEF, left ventricular ejection fraction; LVIDd, left ventricular internal diameter end-diastole.
